# A Qualitative Analysis of Provider-Level Barriers to Prescribing Diabetes Technology

**DOI:** 10.1155/jdr/8873848

**Published:** 2025-11-04

**Authors:** Jennifer Maizel, Ashby F. Walker, Anna Walls, Francisco J. Pasquel, Michael J. Haller, Brittany S. Bruggeman

**Affiliations:** ^1^Department of Medicine, University of Miami Miller School of Medicine, Miami, Florida, USA; ^2^Department of Health Services Research, Management, and Policy, University of Florida College of Public Health and Health Professions, Gainesville, Florida, USA; ^3^University of Florida College of Medicine, Gainesville, Florida, USA; ^4^Department of Medicine, Division of Endocrinology, Emory University School of Medicine, Atlanta, Georgia, USA; ^5^Department of Pediatrics, Division of Endocrinology, University of Florida College of Medicine, Gainesville, Florida, USA

## Abstract

**Background:**

Patient utilization of diabetes technology differs based on sociodemographic and other factors. Underserved patients have reported that providers decline to prescribe continuous glucose monitors (CGMs) and insulin pumps. This qualitative study elucidated provider perspectives regarding facilitators and barriers to the prescription and patient use of diabetes technology.

**Methods:**

Sixteen diabetes care providers (75.0% MD, 18.8% APRN, 6.3% PharmD, and 50.0% adult endocrinology) at academic health centers, a Veterans Affairs Medical Center, and a safety net hospital in the southeastern United States were surveyed and interviewed from January to May 2024. Survey data were analyzed using descriptive statistics; a thematic analysis was used for interview transcripts with an adapted version of the social-ecological model (SEM) as the coding framework.

**Results:**

On the survey, providers estimated that 80% (IQR 58.0%–86.0%) of their patients who met American Diabetes Association criteria for CGMs and 50% (IQR 48.0%–63.0%) for insulin pumps regularly used them. System factors (e.g., lack of insurance, high device costs, and insurance bureaucracy) were perceived by providers (62.5%–93.8%) as patients' top barriers to use. Across the interviews (*n* = 362 codes), providers' top prescribing barriers were also system-level (65.7%), including working with insurance, durable medical equipment (DME) companies, and pharmacies (15.7%), checking eligibility requirements (11.0%), and electronic health record (EHR) limitations (9.7%). Interpersonal prescribing barriers (7.5%) were tied to patients having low health literacy (3.6%) and communication with non–English-speaking patients (1.7%). Individual prescribing barriers (26.0%) included patients expressing concerns about device adhesives/appearance (5.5%) and patients having limited knowledge/interest (3.0%). Facilitators across SEM levels included simplified eligibility criteria, EHR order sets, shared decision-making, and proactive insurance/DME companies.

**Conclusions:**

These findings indicate the need for multilevel solutions to improve the prescription and use of diabetes technology. Future research and clinical practice should aim to enhance EHR functionality and system integration, improve patient-provider communication, and streamline insurance criteria and processes.

## 1. Introduction

The development and evolution of diabetes technology over the past 20 years, including insulin pumps, continuous glucose monitors (CGMs), and artificial insulin delivery (AID) systems, have revolutionized diabetes care and resulted in improved HbA1c, time in range (TIR), rates of hypoglycemia, and quality of life among people with insulin-dependent diabetes [1–4]. Encouragingly, individuals with the highest HbA1c levels have the greatest improvement in these measures when starting diabetes technology [5]. However, socioeconomic and racial/ethnic inequities in diabetes technology use are evident across all age groups and prevent some of the most underserved populations from benefitting [6–8]. Between 2010 and 2012 and 2016 and 2018, CGM use increased by 41% in the highest socioeconomic status (SES) quintile but by only 12% in the lowest; similar differences exist in insulin pump use, and these correlate with disparities in HbA1c [9]. Even after adjusting for SES and other factors, racial/ethnic inequities persist in diabetes technology use, with non-Hispanic (NH) Black patients having lower utilization than NH White patients [6–8].

Provider-level factors, including providers declining to prescribe diabetes technology, were reported by underserved patients with Type 1 diabetes (T1D) as being key barriers to insulin pump and CGM use in several recent studies [10–15]. Diabetes care providers are often patients' main source of information regarding diabetes treatment options and the primary resource for supplying and training patients on diabetes technologies [16]. Negative patient-provider interpersonal interactions, a lack of shared decision-making, unclear communication surrounding eligibility criteria, and negative provider attitudes regarding diabetes devices have been reported as barriers to technology access among underserved groups [10–15]. Similarly, providers' decisions regarding whether to prescribe diabetes technology emerge from a combination of individual-, interpersonal-, and system-level factors in which providers operate [17–19]. While studies have evaluated perceived facilitators and barriers to use of diabetes technology by underserved patients [10–15], to our knowledge, there have been no US-based studies employing semistructured interview methodology to investigate the perspectives of diabetes care providers.

The aim of the present study was to determine facilitators and barriers to the prescription and patient use of diabetes technology from the provider's perspective, both generally and within underserved populations. To address this aim, we employed qualitative and descriptive approaches to capture the nuanced perspectives of diabetes care providers. Our study was based on the social-ecological model (SEM), which asserts that an individual's health occurs at and results from factors across multiple levels that include the individual, interpersonal, community, and societal/policy levels [17, 20]; as such, this model provided a comprehensive framework for understanding facilitators and barriers to the prescription and patient use of diabetes technology.

## 2. Materials and Methods

### 2.1. Study Design

Utilizing surveys and semistructured interviews, we analyzed diabetes care providers' perspectives regarding facilitators and barriers to the prescription of insulin pumps and CGMs, both generally and specifically for underserved patients. This study was part of a larger ongoing research effort to develop an electronic health record (EHR) dashboard aimed at improving the prescription of insulin pumps and CGMs for underserved patients with diabetes.

### 2.2. Participants

We surveyed and interviewed diabetes care providers treating pediatric and adult patients with T1D as well as other diabetes types. Providers were recruited via a purposeful and convenience sampling approach from the University of Florida (UF) and Emory University academic health centers, as well as the Malcolm Randall Department of Veterans Affairs Medical Center in Gainesville, Florida, and the Grady Health System Diabetes Center in Atlanta, Georgia. Pediatric and adult endocrinologists, diabetologists, and advanced practice providers working within a diabetes clinic setting were targeted for recruitment. Providers who met the following eligibility criteria were included: (a) provided diabetes care to at least 10 patients with T1D per year, (b) had the ability to prescribe insulin pumps and CGMs, and (c) utilized an EHR platform for their patients. All study procedures were approved by the UF, Emory University, and Nova Southeastern University Institutional Review Boards.

### 2.3. Procedure

#### 2.3.1. Data Collection

Guided by the principle of data triangulation [21], our study employed both surveys and semistructured interviews to cross-validate findings and identify consistent patterns and themes. All surveys and interviews were completed between January 2024 and May 2024. Upon agreement to participate in this study, providers were emailed a link to complete the survey. The survey was administered via Research Electronic Data Capture (REDCap, Nashville, TN), an online data management platform. The survey confirmed providers met the study eligibility criteria, captured their demographic/professional information, and collected information regarding their patient panels. Surveys were completed anonymously without any linked identifying information. The survey also presented providers with a list of 19 anticipated barriers to prescribing CGMs and insulin pumps, based on feedback provided by patients and providers in earlier research [10, 19, 22, 23], and asked providers to indicate which barriers they encountered among their patient panels. Additionally, the survey asked providers to rank their perceived top five barriers and note the “biggest barrier.”

Providers who completed the survey and met eligibility criteria were later contacted via email to schedule a one-on-one interview. Interviews were conducted via Zoom (Zoom Communications, San Jose, California), a videoconferencing platform, and lasted between 30 and 60 min. Interview questions (Table 1) were written by the research team to align with the specific aims of this study as well as the broader research goal to develop an EHR dashboard for prescribing diabetes technology. Interview questions focused mainly on T1D populations but could also include discussions surrounding Type 2 diabetes (T2D) populations if relevant. Interview questions were also modeled after those included in an interview protocol with similar objectives [10]. Interviews followed a semistructured format; all participants were asked a standardized set of questions, and follow-up and probe questions were asked as needed for clarification and/or to solicit deeper insights. Informed by reflexivity [24], the lead author conducted the interviews, drawing on expertise in behavioral science and diabetes care. Additionally, the interviewer's nonclinical role helped create an open, conversational atmosphere in which participants felt comfortable expressing their perspectives in depth.

#### 2.3.2. Data Analysis

Survey data were analyzed using descriptive statistics (*n*, %, median, and interquartile range [IQR]). All survey questions were optional; as such, the number of responses to some questions varied. Percentages were calculated out of the total number of participants, even if some responses were missing (e.g., a provider ranked <5 top barriers) (see Tables 2, 3, and 4). All survey data analysis was completed in Microsoft Excel (Microsoft, Redmond, Washington).

Interview recordings were transcribed by DataGain, an external qualitative analysis vendor. Interview transcripts were also analyzed by two DataGain coders in MAXQDA (VERBI Software, Berlin) using a deductive thematic analysis approach. Only interview data pertaining to facilitators and barriers to prescribing diabetes technologies were included in this study. An adapted version of the SEM served as the guiding thematic analysis framework. We adapted the SEM using a coding schematic with categories and subcategories that included the following: (a) individual-level facilitators and barriers related to patients' and providers' personal values, beliefs, preferences, and sociodemographic characteristics; (b) interpersonal-level facilitators and barriers related to interactions between patients, providers, and/or health centers; and (c) system-level facilitators and barriers related to EHR platforms, paperwork and bureaucracy, broader cultural and economic factors, and policies from other healthcare system players such as insurance companies, pharmacies, and durable medical equipment (DME) suppliers.

The first coder had expertise in public health, healthcare management, and quality improvement. They were involved in the project early on and developed a thematic analysis and codebook through an iterative process with frequent feedback from the study investigators. The established codebook served as the framework for subsequent analysis conducted by the second coder. To ensure consistency in the two coders' application of the coding scheme, interrater reliability (IRR) was calculated for a 35% subset of the transcripts in MAXQDA. Specifically, the MAXQDA “Code Occurrence in the Document” function was used to determine coders' percent agreement for each selected code in each line of the interview transcripts, as well as their mean percent agreement across all codes. Coding discrepancies were resolved through discussion and consensus.

## 3. Results

### 3.1. Survey Results

#### 3.1.1. Providers' Demographic Characteristics and Patient Panel Information

Sixteen providers participated in this study. The majority were female (*n* = 10, 62.5%), White (*n* = 10, 62.5%), had a median age of 39 years (IQR = 36 − 40.5 years), earned a Medical Doctor (MD) degree (*n* = 12, 75.0%), practiced adult endocrinology (*n* = 8, 50.0%), practiced for a median of 6 years (IQR = 3.5 − 9.25 years), and were currently employed at an academic medical center (*n* = 15, 93.8%). These providers' demographics did not differ substantially from the 50 providers who were approached for recruitment, who were majority female (*n* = 34, 68%), who earned a MD degree (*n* = 34, 68%), and with around half practicing adult endocrinology (*n* = 22, 44%).

On average, the participating providers reported that they saw 35 (IQR = 24 − 46) patients per week, of whom 23 (IQR = 10 − 30) had diabetes. Additionally, they estimated that 90% (IQR = 80% − 91%) of their patients with diabetes required insulin therapy, 45% (IQR = 30% − 80%) of their patients with diabetes had T1D, 80% (IQR = 58% − 86%) of their patients who qualified for CGM devices based upon the American Diabetes Association (ADA)'s 2024 Standards of Care criteria regularly used them, and 50% (IQR = 48% − 63%) of their patients who qualified for insulin pumps based upon the ADA's 2024 Standards of Care criteria regularly used them [23].

The participating providers estimated that 40% (IQR = 23% − 60%) of their patients used Medicaid as their primary insurance, 20% (IQR = 0% − 45%) of their patients used Medicare as their primary insurance, and 5% (IQR = 2% − 10%) of their patients were uninsured, with the remaining covered by private insurance.  Table 2 presents all participating providers' demographic characteristics and patient panel summary information.

#### 3.1.2. Barriers to Diabetes Patients' Usage of CGM Devices From the Provider Perspective

On the provider survey, the five most frequently reported barriers to qualifying diabetes patients' regular usage of CGM devices were as follows: (a) lack of insurance or excessive copays/coinsurance (*n* = 15, 93.8%), (b) cost of device (*n* = 12, 75.0%), (c) excessive bureaucratic barriers to obtaining insurance coverage (*n* = 12, 75.0%), (d) excessive bureaucratic barriers to determining the appropriate pharmacy vs. DME supplier (*n* = 10, 62.5%), and (e) patient/caregiver being unable to keep device on skin (*n* = 9, 56.3%). Furthermore, lack of insurance coverage or excessive copays/coinsurance was most frequently reported by providers as the “biggest barrier” (*n* = 7, 43.8%). Cost of device was the second most frequently reported “biggest barrier” (*n* = 4, 25.0%). Interestingly, no providers reported provider-related factors as barriers. Over a third of providers reported that patient and caregiver inability to understand or use technology effectively was a barrier to regular use, with one-quarter of providers identifying this as a top five barrier.  Table 3 presents all barriers to qualifying diabetes patients' regular usage of CGM devices, as indicated by providers on the survey.

#### 3.1.3. Barriers to Diabetes Patients' Usage of Insulin Pumps From the Provider Perspective

Regarding qualifying diabetes patients' regular usage of insulin pumps, the five most frequently reported barriers on the provider survey were nearly the same as those reported for CGM devices: (a) lack of insurance coverage or excessive copays/coinsurance (*n* = 14, 87.5%), (b) cost of device (*n* = 12, 75.0%), (c) excessive bureaucratic barriers to obtaining insurance coverage (*n* = 10, 62.5%), (d) patient/caregiver does not want/like having diabetes devices on their body (*n* = 9, 56.3%), (e) patient/caregiver is not interested in an insulin pump (*n* = 8, 50.0%), and (f) excessive bureaucratic barriers to determining appropriate pharmacy vs. DME supplier (*n* = 8, 50.0%). Additionally, lack of insurance coverage or excessive copays/coinsurance was reported as the “biggest barrier” for qualifying diabetes patients' regular usage of insulin pumps (*n* = 7, 43.8%), followed by the cost of the device (*n* = 5, 31.3%). In contrast to their responses regarding CGM barriers, one provider (6.3%) did note a provider-related knowledge factor as a barrier to the usage of insulin pumps.  Table 4 presents all provider-reported barriers to qualifying diabetes patients' regular usage of insulin pumps, as indicated on the survey.

### 3.2. Semistructured Interview Results

**Figure 1:**
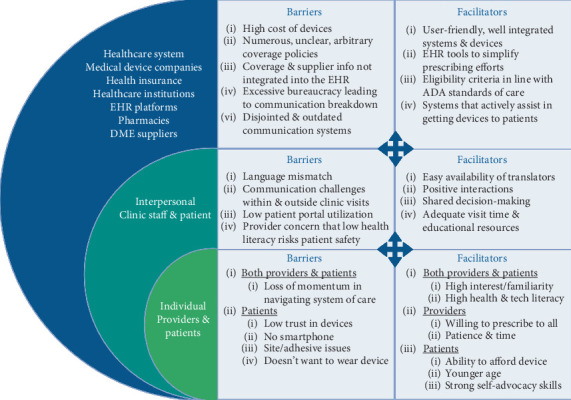
Social-ecological model of facilitators and barriers to prescription and use of diabetes technology, including insulin pumps, continuous glucose monitors (CGMs), and artificial insulin delivery systems (AIDs). Results are based upon survey and semistructured interview data from 16 diabetes care providers. Barriers and facilitators at one level interact with and can either compound upon or ameliorate those of a different level, further driving disparities in access. ADA = American Diabetes Association; DME = durable medical equipment; EHR = electronic health record.

A total of 362 codes across 30 subthemes related to diabetes technology prescribing facilitators and barriers were identified in the provider interview transcripts. IRR between the two transcript coders was strong (mean % agreement across all codes = 90%). Subthemes linked to the system level of the adapted SEM were the most frequently coded (*n* = 238 codes, 65.7%); the three codes with the highest occurrence were (a) system factors: health insurance companies, DME suppliers, and pharmacies (*n* = 57 codes, 15.7%), (b) system factors: criteria/requirements for coverage (*n* = 40 codes, 11%), and (c) system factors: EHR solutions and recommendations (*n* = 35 codes, 9.7%). Twenty-seven codes were related to interpersonal factors (7.5%) and 94 related to individual factors (26%). Findings from the thematic analysis are summarized in the following sections according to each adapted SEM level (system, interpersonal, and individual levels) and presented in Figure 1. Example interview quotes regarding facilitators and barriers occurring at each adapted SEM level are presented in Table 5.

#### 3.2.1. System-Level Facilitators and Barriers

System-level facilitators and barriers discussed during the provider interviews reflected the overarching healthcare system as well as subsystems within it (e.g., the health insurance marketplace, medical device companies that produce insulin pumps and CGMs, DME suppliers, pharmacies, healthcare institutions/clinics, clinical care teams, and EHR platforms).

Providers asserted that facilitators at the system level included patients having DME suppliers that proactively assist them with getting their devices; recent changes in Medicaid eligibility criteria that increase certain patients' likelihood of being prescribed diabetes technologies (e.g., patients identified as “at risk for hypoglycemia” having a greater chance of receiving CGM approval); and providers having EHR platforms that simplify diabetes technology prescribing efforts (e.g., EHRs have predetermined order sets that match insulin pump cartridges, syringes, and tubing, and/or EHRs display patients' insurance coverage information). Furthermore, providers noted that a key facilitator at the system level relates to medical device companies creating insulin pumps and CGMs that are well-marketed, user-friendly, compatible with patients' smartphones, and integrated with EHR platforms.

Health insurance stipulations (e.g., high patient copays, high deductibles, and arbitrary eligibility criteria) were identified by providers as pervasive system-level barriers to prescribing diabetes technologies. Providers described how continually changing insurance policies are a source of frustration for them, especially since some described that they are not notified of these changes in a timely manner or at all. They also mentioned substantial differences in eligibility criteria between insurance companies that require them to invest significant time into learning and keeping up with insurance information. Examples described included insurance companies requiring patients to have a minimum diabetes duration, proven attendance at diabetes education classes, proven attendance at follow-up clinic visits, and/or a maintained log of blood glucose values to receive coverage for insulin pumps and CGMs. Providers noted numerous barriers specific to Medicaid [25] (e.g., certain state Medicaid policies do not cover CGMs for adult patients and/or do not cover diabetes education classes that teach patients how to use these devices). Other system-level barriers included excessive paperwork for prescriptions and prior authorizations, challenges with following up with DME suppliers and pharmacies to ensure prescriptions were filled, and the inability to use EHR platforms to submit prescriptions to DME suppliers. Providers described the need to scan and fax prescriptions, adding time and opportunities for communication lapses. Providers also noted complexities with prescribing the devices themselves (e.g., certain insulin pumps and CGMs are made up of multiple components, all of which need to be prescribed separately). Furthermore, incompatibility between certain devices and components with patients' existing devices and/or smartphones was identified as a barrier.

#### 3.2.2. Interpersonal-Level Facilitators and Barriers

Providers mentioned an array of interpersonal-level facilitators and barriers associated with prescribing insulin pumps and CGMs; these pertained to interactions between patients, providers, and clinic staff. Patients' health literacy (*n* = 13, 3.6% of codes) and primary language spoken (*n* = 6, 1.7% of codes), as well as the quality of their interactions with providers and clinic staff, were identified as contributing factors.

Facilitators included positive patient-provider interactions; shared decision-making in diabetes care between patients and providers; the presence of in-person, human language translators during clinic visits; providers having more time and resources available to communicate with patients about the types and value of diabetes technologies; and patients demonstrating strong self-advocacy skills when navigating healthcare resources.

For interpersonal-level barriers, nearly half of the interviewed providers (*n* = 7; 43.8%) described communication challenges with patients who had low health literacy and numeracy (e.g., patients who were homeless, had intellectual disabilities, and/or spoke a language other than English). Most providers stated that they initiate a conversation regarding the benefits of diabetes technology with all their eligible patients and prescribe the devices to all patients who express interest, but some felt that health literacy barriers and associated risks to patient safety warranted the decision not to prescribe (*n* = 7; 43.8%). Examples included concerns regarding patients' inability to monitor their symptoms consistently, follow instructions, and understand and utilize math skills. This especially impacted their decision to prescribe insulin pumps.

Providers noted that while having in-person language translators attend clinic visits is valuable, doing so requires more time. Furthermore, they described how the nuances of diabetes management and technologies can be challenging to accurately translate. Lastly, they mentioned that older patients, older caregivers, and Spanish-speaking patients had lower utilization of patient portals (e.g., MyChart); this created barriers for communicating with patients and caregivers between/outside of clinic visits.

#### 3.2.3. Individual-Level Facilitators and Barriers

In the interviews, providers noted that individual-level facilitators and barriers to prescribing diabetes technologies primarily focused on personal values, knowledge, and preferences held by patients and providers, as well as patients' sociodemographic characteristics.

Among patients, individual-level facilitators as reported by providers included greater interest in using insulin pumps or CGMs (*n* = 11, 3.0% of codes), ability to afford the devices, higher health literacy, and greater savviness/comfort with using technology. Providers mentioned that younger patients were typically more comfortable using devices than older patients. Among providers, individual-level facilitators included high conscientiousness toward patients, high familiarity with various insulin pump and CGM devices and brands, and patience/willingness to take the necessary time to explain device-related information to patients. Providers described how high conscientiousness in this context manifests as a willingness to prescribe diabetes technologies to “everyone,” especially patients who have suboptimal glycemic control (e.g., higher HbA1c values). Providers described that, as they were diabetes specialists, they had high knowledge of insulin pumps and CGM devices, as well as the health insurance complexities associated with prescribing them.

Individual-level barriers among patients as reported by providers included limited knowledge about insulin pumps and CGMs, lower trust in medical devices, not having a smartphone, not being able to afford the devices, and having concerns about wearing medical devices (e.g., potential adverse skin reactions, the devices peeling or falling off, and feelings that wearing the devices signified a constant reminder about having diabetes). Providers noted the latter concern was more frequently expressed by adolescent patients. Additionally, providers mentioned that concerns about wearing devices (*n* = 20, 5.5% of codes) more commonly affected patients with labor-intensive occupations, such as construction workers. Lastly, “loss of momentum,” referring to delays between a patient expressing interest in a device and later picking up/getting the prescription, was noted as an individual-level barrier, although it also related to factors at the interpersonal and system levels. This loss of momentum was attributed to a variety of factors, including patients lacking access to transportation and/or being unable to take time off from work.

Providers speculated that individual-level barriers for providers were more applicable to primary care providers (e.g., having less knowledge regarding the devices and insurance challenges). They also noted that older providers may encounter individual-level barriers such as lower comfort with technology and imposing stricter and/or outdated prescribing criteria on their patients (e.g., requiring patients to have a particular HbA1c or body mass index [BMI] to be prescribed a device). One provider mentioned provider implicit bias toward patients from marginalized backgrounds as another individual-level barrier.

## 4. Discussion

To our knowledge, our study is the first US-based effort employing rigorous interview methodology to identify diabetes technology prescription facilitators and barriers from the perspective of diabetes care providers. In our study, providers reported a multitude of facilitators and barriers occurring at all levels of our adapted SEM framework. Overall, system-level barriers were the most frequently reported. Many facilitators and barriers also interacted across levels. It is important to highlight providers' notion of “lost momentum,” between when a patient first expresses interest in a device and actually begins using it. This finding elucidates the interconnectedness of facilitators and barriers at each level of the SEM, which can increase or decrease inequities in technology usage. For example, patients with greater willingness to use the devices but lower health literacy (individual-level factors), as well as a language barrier (interpersonal-level factor), may have a lower ability to navigate the challenges of communication breakdowns between systems of care (e.g., clinics, insurance companies, and DME suppliers [system-level factors]).

### 4.1. System-Level Implications

System-level barriers such as high device costs, ever-changing and unclear health insurance coverage, and DME supplier policies, as well as poor EHR integration, pervaded providers' survey and interview responses. Prior research has examined insurance-related barriers and defined provider insurance bias as clinicians recommending more technology to patients with private insurance compared to patients with public insurance [26]. Our study demonstrates that provider biases tied to insurance coverage are rooted in systemic barriers, including arbitrary and outdated coverage policies that vary by insurance and the inordinate amount of time providers and staff must take to complete prior authorizations and utilize outdated communication systems beyond their regular patient care responsibilities. This finding is further supported by a 2024 American Medical Association survey of 1000 physicians, which found that practices spend an average of 13 h/week completing prior authorization paperwork, and physicians complete an average of 39 prior authorizations per week [27].

Our study also elucidates insights into system-level barriers and facilitators that have not been focus areas of prior research, especially those pertaining to DME companies and EHR functionality. Some provider-level barriers previously reported by patients [10–15], including unclear prescription eligibility criteria and providers' negative views of diabetes technology, may actually be rooted in these system-level barriers. Additionally, system-level facilitators reported by providers in our study, such as simplified device eligibility criteria based on ADA's current Standards of Care [28], EHR order sets, and health insurance and DME companies actively facilitating each step of the prescription, coverage, and delivery process, present opportunities to ameliorate system-level barriers. For example, interventions to improve EHR integration, modernize communication systems, and expand and streamline health insurance coverage should be studied as potential interventions to improve diabetes technology prescription and use.

### 4.2. Interpersonal-Level Implications

Despite system-level barriers being the most frequently reported in our study, our findings also indicate a need to address barriers occurring at other SEM levels. At the interpersonal level, patient-provider communication challenges were paramount issues affecting providers' diabetes technology prescribing decisions. Although providers in our study did not report establishing glycemic control parameters for prescribing diabetes technology, several felt that communication challenges with patients who had low health literacy or spoke a different language reduced their likelihood of prescribing diabetes technology. As such, interventions aimed at promoting positive and comprehensive interactions between patients, providers, and clinics are needed. For example, communication models that employ shared decision-making regarding diabetes technology have been shown to attenuate racial/ethnic disparities in diabetes technology use that can occur when providers make decisions unilaterally [12]. Recent research has also shown that connecting underserved patients with peer mentors who share lived experience with diabetes can increase their utilization of CGMs [29]. Moreover, as noted by providers in this study, the utilization of in-person language translators during clinic visits presents opportunities to improve diabetes technology prescribing and patient usage.

### 4.3. Individual-Level Implications

Furthermore, our study demonstrated that individual-level factors, such as patient interest in devices and health literacy, and provider biases and conscientiousness, directly affect device prescribing and patient usage as well as frequently interact with system- and interpersonal-level barriers to either exacerbate or ameliorate disparities in diabetes technology use. Providers in our study reported higher estimated regular patient usage of CGM than usage of insulin pumps. “Patient not interested” was a top-five barrier for insulin pump use but not CGM use. Taken together, these findings indicate that CGM is increasingly viewed as the standard of care, while patient preference still plays a key role in insulin pump prescribing and usage. When the ADA's Standards of Care first published a “Diabetes Technology” section in 2019 [30], they recommended T1D treatment with either multiple daily injections (MDIs) or an insulin pump. Offering insulin pumps and AID systems was first strongly recommended for T1D patients in 2022 [31], and ADA's latest 2025 Standards of Care are the first to state that AID systems should be the preferred insulin delivery method for those with T1D, with early initiation recommended [28]. ADA's 2025 Standards of Care are also the first to state that insulin pumps should be offered to T2D patients on MDI [28]. Efforts should be made to publicize these recommendations, as our results indicate that following them will require a mindset shift, even for specialized academic diabetes care providers, and will result in improved glycemic control and quality of life for patients [32, 33].

Previous research has shown that subjective factors, including perceptions of whether patients possess the personal and psychological attributes needed to use diabetes technology, often influence provider prescriptions [34]. The ADA's Standards of Care have reinforced this view in the past, stipulating that perceived benefits of technology correlate with regular utilization, and these perceived benefits are only present with proper usage [30]. Furthermore, previous iterations specified that providers should account for an individual patient's skill level when deciding whether to offer diabetes technology [35]. A shift in the 2022 Standards of Care indicated that only the choice of device, rather than offering technology at all, should be influenced by skill level [31], and the 2025 Standards of Care eliminated the recommendation that CGM only be offered to individuals who “are capable of using the device” [28]. This recommendation persists for AID systems. As more studies demonstrate the safe use of AID systems even in high-risk groups, this paradigm may be reconsidered [36–39]. Previous studies have also demonstrated that viewing insulin pumps as just another care delivery system rather than as a liability increases overall clinic prescription and patient use of this technology [40]. Additionally, seeing patients perceived as having high safety risks improve their outcomes with AID systems can improve providers' perspectives on prescribing technology to all [41, 42].

### 4.4. Future Directions

Our findings indicate the need for multilevel interventions to improve the prescription and patient usage of diabetes technology. This study was part of a larger ongoing system-level research effort to develop an EHR dashboard aimed at improving the prescription of insulin pumps and CGMs for underserved patients. Other potential interventions could aim to reduce insurance complexities, promote shared decision-making between patients and providers, increase patients' comfort with using devices, and improve providers' perceptions. Future studies should assess diabetes technology prescription facilitators and barriers among primary care providers, who face additional burdens regarding time, dedicated resources, and specialty-specific knowledge [43], as well as diabetes care providers in other regions of the United States and internationally. Such studies would yield insights into state and national policy differences. For example, a recent study of adults with diabetes receiving care at Federally Qualified Health Centers in Florida and California found that patients' insurance coverage and technology usage varied by state, with Florida having higher rates of uninsured and lower CGM and pump use [44]. Additionally, further research should determine whether there are evidence-based factors that should be considered when assessing the capability of a patient to use diabetes technology.

### 4.5. Strengths and Limitations

Our study's strengths lie in its structured and comprehensive analysis of facilitators and barriers to the prescription of diabetes technology from the perspective of diabetes care providers. The providers included in our study reported a high percentage of patients with Medicaid and Medicare insurance and a mix of pediatric, adult, T1D, and T2D patients. Other studies have also found system-level barriers that interact with interpersonal and individual factors that influence the prescription of diabetes technology [22, 45]. Our findings build upon this previous work by identifying unique system-level barriers, including poor DME supplier integration into EHR systems and other EHR constraints. Limitations of our study include the potential for various biases (e.g., survey recall bias, observer bias, and self-selection bias), its small sample size, and its focus on diabetes providers employed at academic health centers in the southeastern United States, for which empirical patient data could also be matched in the future. These limitations may reduce the generalizability of our findings to other geographic areas; similarly, specific aspects of the US healthcare system limit generalizability to areas outside of the United States.

## 5. Conclusions

Our study highlights the need for multilevel improvements, especially at the system level, to enable and empower diabetes care providers to prescribe diabetes technology to underserved patients and more broadly. Future studies and interventions should focus on improving EHR functionality and integration across systems, simplifying insurance eligibility criteria, enhancing patient-provider communication, and reducing challenges with device adhesives and wearability, with the ultimate aim of providing accessible diabetes technology to all who can benefit.

## Figures and Tables

**Table 1:** Subset of provider semistructured interview questions regarding diabetes technology prescription facilitators and barriers.

1. What are facilitators and barriers to prescribing insulin pumps and CGMs to underserved populations?
2. Among the barriers you identified, which ones are worse for underserved populations such as individuals with lower incomes, enrolled in Medicaid, and racial/ethnic minorities? Why?
3. Do you or your clinic have any specific requirements that need to be met prior to patients being prescribed a CGM? What about for insulin pumps?
4. Considering the barriers that we just discussed, what functionalities or tools in the EHR, if any, make prescribing diabetes technologies easier?
5. What changes to the EHR's tools and functionalities would you recommend to make prescribing diabetes technologies easier?
6. Lastly, is there anything else you would like to share regarding facilitators and unforeseen barriers to diabetes technology prescriptions for underserved populations?

Abbreviations: CGM = continuous glucose monitor; EHR = electronic health record.

**Table 2:** Providers' demographic characteristics and patient panel information (*n* = 16, unless otherwise specified).

**Provider demographic characteristics**	
Gender	*n* (%)
Female	10 (62.5)
Male	5 (31.3)
Unspecified	1 (6.3)
Race/ethnicity	*n* (%)
White	10 (62.5)
Hispanic/Latino	5 (31.3)
Asian	2 (12.5)
Age in years (*n* = 15)	Median (IQR) 39 (36–40.5)
Healthcare degrees/credentials	*n* (%)
MD	12 (75.0)
APRN	3 (18.8)
PharmD	1 (6.3)
Practice area/specialty	*n* (%)
Adult endocrinology	8 (50.0)
Pediatric endocrinology	6 (37.5)
General internal medicine	1 (6.3)
Unspecified	1 (6.3)
Years in practice	*n* (%)
0–2 years	4 (25.0)
3–6 years	4 (25.0)
7–10 years	5 (31.3)
>10 years	3 (18.8)
Practice type	*n* (%)
Academic medical center	15 (93.8)
Hospital-owned medical group	1 (6.3)
# of diabetes care providers in the same practice	Median (IQR) 13.5 (9–21.25)
Patient panel information	Median (IQR)
# of patients seen per week	35 (24–46)
# of patients with diabetes seen per week	23 (10–30)
% of patients with T1D	45 (30–80)
% of patients requiring insulin therapy	90 (80–91)
% of qualified patients regularly using CGM devices	80 (58–86)
% of qualified patients regularly using insulin pumps	50 (48–63)
% of patients with medicaid insurance (*n* = 15)	40 (23–60)
% of patients with medicare insurance (*n* = 15)	20 (0–45)
% of uninsured patients (*n* = 15)	5 (2–10)

Abbreviations: APRN = advanced practice registered nurse; CGM = continuous glucose monitor; IQR = interquartile range; MD = Medical Doctor; PharmD = Doctor of Pharmacy; T1D = Type 1 diabetes.

**Table 3:** Providers' perceived barriers to diabetes patients' regular usage of CGM systems and perceived “biggest barrier” (*n* = 16)^a^.

**Perceived barriers to CGM use**	**n** ** (%)**	**Top 5 barriers, ** **n** ** (%)**	**“Biggest barrier,” ** **n** ** (%)**
Lack of insurance coverage despite meeting eligibility or excessive copays/coinsurance	15 (93.8)	11 (68.8)	7 (43.8)
Cost of device	12 (75.0)	8 (50.0)	4 (25.0)
Excessive bureaucratic barriers to obtaining insurance coverage (e.g., prior authorizations or other paperwork)	12 (75.0)	12 (75.0)	2 (12.5)
Excessive bureaucratic barriers to determining the appropriate pharmacy vs. DME supplier	10 (62.5)	8 (50.0)	0 (0.0)
Patient/caregiver unable to keep device on skin	9 (56.3)	4 (25.0)	0 (0.0)
Patient/caregiver does not want/like having diabetes devices on their body	8 (50.0)	4 (25.0)	0 (0.0)
Patient/caregiver concern about device accuracy and/or functioning	7 (43.8)	4 (25.0)	0 (0.0)
Patient/caregiver is not interested in CGM	6 (37.5)	5 (31.3)	1 (6.3)
Patient/caregiver unable to understand or use technology effectively	6 (37.5)	4 (25.0)	1 (6.3)
Patient/caregiver skin reactions or pain	5 (31.3)	2 (12.5)	0 (0.0)
Too many alarms	5 (31.3)	2 (12.5)	0 (0.0)
Lack of clinic resources to educate patients on CGM	4 (25.0)	4 (25.0)	0 (0.0)
Lack of clinic infrastructure to use CGM data	4 (25.0)	2 (12.5)	0 (0.0)
Lack of access to CGM samples in clinic	4 (25.0)	2 (12.5)	0 (0.0)
Lack of time in clinic to discuss CGM	3 (18.8)	2 (12.5)	0 (0.0)
Patient/caregiver does not want to take more time from their day to use a CGM	1 (6.3)	0 (0.0)	0 (0.0)
Provider needs more knowledge about CGM placement and day-to-day use	0 (0.0)	0 (0.0)	0 (0.0)
Provider needs more knowledge about interpretation of CGM data	0 (0.0)	0 (0.0)	0 (0.0)
Interferes with sleep	0 (0.0)	0 (0.0)	0 (0.0)

Abbreviations: CGM = continuous glucose monitor; DME = durable medical equipment.

^a^Calculated out of *n* = 16 providers despite one provider providing incomplete responses to some barrier-related survey questions.

**Table 4:** Providers' perceived barriers to qualifying diabetes patients' regular usage of insulin pumps, including perceived “biggest barrier” (*n* = 16)^a^.

**Perceived barriers to insulin pump use**	**n** ** (%)**	**Top 5 barriers, ** **n** ** (%)**	**“Biggest barrier,” ** **n** ** (%)**
Lack of insurance coverage despite meeting eligibility, excessive copays, or coinsurance	14 (87.5)	12 (75.0)	7 (43.8)
Cost of device	12 (75.0)	11 (68.8)	5 (31.3)
Excessive bureaucratic barriers to obtaining insurance coverage (e.g., prior authorizations or other paperwork)	10 (62.5)	9 (56.3)	1 (6.3)
Patient/caregiver does not want/like having diabetes devices on their body	9 (56.3)	9 (56.3)	1 (6.3)
Patient/caregiver is not interested in an insulin pump	8 (50.0)	8 (50.0)	1 (6.3)
Excessive bureaucratic barriers to determining the appropriate pharmacy vs. DME supplier	8 (50.0)	7 (43.8)	0 (0.0)
Patient/caregiver unable to understand or use technology effectively	4 (25.0)	5 (31.3)	0 (0.0)
Lack of clinic resources to educate patients on insulin pump use	4 (25.0)	3 (18.8)	0 (0.0)
Patient/caregiver concern about device accuracy and/or functioning	4 (25.0)	2 (12.5)	0 (0.0)
Patient/caregiver unable to keep device on skin	4 (25.0)	2 (12.5)	0 (0.0)
Lack of time in clinic to discuss insulin pumps	2 (12.5)	2 (12.5)	0 (0.0)
Lack of clinic infrastructure to use insulin pump data	2 (12.5)	2 (12.5)	0 (0.0)
Patient/caregiver skin reactions or pain	2 (12.5)	1 (6.3)	0 (0.0)
Patient/caregiver does not want to take more time from their day to use an insulin pump	2 (12.5)	1 (6.3)	0 (0.0)
Too many alarms	2 (12.5)	0 (0.0)	0 (0.0)
Lack of access to insulin pump demo samples in clinic	1 (6.3)	1 (6.3)	0 (0.0)
Provider needs more knowledge about insulin pump placement and day-to-day use	1 (6.3)	0 (0.0)	0 (0.0)
Provider needs more knowledge about interpretation of insulin pump data	0 (0.0)	0 (0.0)	0 (0.0)
Interferes with sleep	0 (0.0)	0 (0.0)	0 (0.0)

Abbreviation: DME = durable medical equipment.

^a^Calculated out of *n* = 16 providers despite one provider providing incomplete responses to some barrier-related survey questions.

**Table 5:** Understanding facilitators and barriers to the prescription of diabetes technologies for underserved diabetes patients using an adapted version of the social-ecological model (SEM).

**SEM level**	**Facilitators/barriers**	**Example provider quote(s)**
System level	Facilitators	“But with the appropriate documentation and assistance from the pump representatives in the area, I think that we have been quite successful at getting people on insulin pumps.” “Care sets, we have developed care sets with trying to pair the right glucometer with the right lancets, right strips. And also if you are ordering of say a CGM that has multiple components, okay, grouping them like this model with this transmitter, with this sensor, with this reader. So that's what has helped our practice in my case a lot.”
System level	Barriers	“For many patients even when they have an insurance, it's not necessarily a 90% coverage, you still have to pay 50% of the cost. So even with insurance, there is still the financial barrier.” “Medicaid as it's offered in the State of Florida is that it does not cover diabetes education. So that's been a pretty major barrier that people who already are more likely to be less health savvy, less technology savvy, and also have less health literacy are now also being deprived of an opportunity to increase all those things.” “Another part of it is - so actually our clinic has gone away from using that particular company and trying to put a little more of the burden of using a DME company on the patient. And this has been met with some resistance from some patients because they do not understand their insurance. They do not really understand DME companies. They do not want to really, do all the leg work. And some people's opinion, this should be part of going to the doctor's office and getting an insulin pump or CGM is that they help you get it prescribed, not that they have to call their insurance, spend time on the phone, trying to figure out, all these new terms and then from there, telling them that they want to order this device and then we get the forms we have to fill them out, send them back. I mean, this is a three-month process is what it turns into.”
Interpersonal level	Facilitators	“There are some patients who still struggle with connecting the pieces even when you show it. But I would say that's more like the exception to the rule. And I think if the time and resources are available to educate the patients that a lot of that gap, even with issues of health literacy like a lot of that gap can be overcome.”
Interpersonal level	Barriers	“From year to year, formulary changes, and it just gets to be a burden on us as the providers … And there's only so much that I can do, because I have so many patients. But there's a lot that we put into the hands of the patients. And if we cannot like handhold them through the process, then it may not always get there. … A lot of the stuff could be eliminated if like the patient would just call and find out like what the requirements are for them, because everybody has such a different plan. But some patients just do not do that.” “I think generally the interaction between us as the clinic, the pharmacy or DME and insurance, that trifecta is really hard for people to know who to ask questions to, who to get refills from. I think oftentimes people run out of supplies or have a pump failure or have a technology issue and do not know who to talk to or reach out to the wrong person first and get a run around. That is hard even if you are someone like me who speaks English, has a medical background. Those are hard institutions to interact with. But I think that that's much harder when you have any kind of barrier.” “I think people who are lower socioeconomic, like, disadvantaged socioeconomically are not going to advocate for themselves as much. And so they are not gonna push for them in the way that someone who is maybe more has higher health literacy and more self agency. They're going to potentially come in and they'll want to really try to get those prior authorizations and advocate for themselves more effectively than I think people who are more disadvantaged and may not even know about the technologies.”
Individual level	Facilitators	“Familiarity with these technologies, access to a smart phone, and then having extra time to really delve into how the technology works.” “But for me [an endocrinologist], I do not think there's anything that I can do to improve how I'm prescribing, because I know when it's needed, I will talk to the patient, and then if it were always covered, I would just order it and it would always be done.”
Individual level	Barriers	“The big barriers are a lot of times the actual education of the family that of their interest in a pump. … the time that it takes to really explain and go through the details of the benefits of the pumps with the family. And then in a separate kind of correlate barrier, sometimes just getting them in the clinic, right? Like, higher, no-show rates or transportation issues or missing work to be able to be there to learn about the pumps are just barriers to even them being there to be in clinic for further discussions and conversations.” “I think there's some bias amongst providers as well. Whereas, our more affluent white families seem to get on to technology and pumps quicker. And I do not know if that's a bias from the provider, just not talking about it or offering it or, is it the family is not requesting it?” “I would presume that there could be barriers of just general racism in terms of providing the right care. So, yeah. But if I have to speak from my own personal perspective, I would think that to me the main driver is the access, the insurance coverage, and having it. I would think that the racism does not play a role in my practice, but I do not know, nobody has audited me to see if I'm prescribing more to certain groups or the others.”

Abbreviations: CGM = continuous glucose monitor; DME = durable medical equipment; SEM = social-ecological model.

## Data Availability

The aggregate data that support the findings of this study are available from the corresponding author upon reasonable request.
